# Adjustment of positive end-expiratory pressure to body mass index during mechanical ventilation in general anesthesia: BodyVent, a randomized controlled trial

**DOI:** 10.1186/s13063-024-08107-8

**Published:** 2024-04-26

**Authors:** Helene Selpien, Christine Eimer, David Thunecke, Jann Penon, Dirk Schädler, Ingmar Lautenschläger, Henning Ohnesorge, Tobias Becher

**Affiliations:** Department for Anesthesiology and Intensive Care Medicine, University Hospital Schleswig-Holstein, Campus Kiel, Arnold-Heller Str. 3, 24105 Kiel, Germany

**Keywords:** General anesthesia, Mechanical ventilation, Failure, Postoperative pulmonary complications, Lung-protective ventilation, Lung aeration score, Lung ultrasound, Lung ultrasound score, Positive end-expiratory pressure, Driving pressure, PEEP, Obesity

## Abstract

**Background:**

In patients requiring general anesthesia, lung-protective ventilation can prevent postoperative pulmonary complications, which are associated with higher morbidity, mortality, and prolonged hospital stay. Application of positive end-expiratory pressure (PEEP) is one component of lung-protective ventilation. The correct strategy for setting adequate PEEP, however, remains controversial. PEEP settings that lead to a lower pressure difference between end-inspiratory plateau pressure and end-expiratory pressure (“driving pressure,” ΔP) may reduce the risk of postoperative pulmonary complications. Preliminary data suggests that the PEEP required to prevent both end-inspiratory overdistension and end-expiratory alveolar collapse, thereby reducing ΔP, correlates positively with the body mass index (BMI) of patients, with PEEP values corresponding to approximately 1/3 of patient’s respective BMI. Thus, we hypothesize that adjusting PEEP according to patient BMI reduces ΔP and may result in less postoperative pulmonary complications.

**Methods:**

Patients undergoing general anesthesia and endotracheal intubation with volume-controlled ventilation with a tidal volume of 7 ml per kg predicted body weight will be randomized and assigned to either an intervention group with PEEP adjusted according to BMI or a control group with a standardized PEEP of 5 mbar. Pre- and postoperatively, lung ultrasound will be performed to determine the lung aeration score, and hemodynamic and respiratory vital signs will be recorded for subsequent evaluation. The primary outcome is the difference in ΔP as a surrogate parameter for lung-protective ventilation. Secondary outcomes include change in lung aeration score, intraoperative occurrence of hemodynamic and respiratory events, oxygen requirements and postoperative pulmonary complications.

**Discussion:**

The study results will show whether an intraoperative ventilation strategy with PEEP adjustment based on BMI has the potential of reducing the risk for postoperative pulmonary complications as an easy-to-implement intervention that does not require lengthy ventilator maneuvers nor additional equipment.

**Trial registration:**

German Clinical Trials Register (DRKS), DRKS00031336. Registered 21st February 2023.

**Trial status:**

The study protocol was approved by the ethics committee of the Christian-Albrechts-Universität Kiel, Germany, on 1st February 2023. Recruitment began in March 2023 and is expected to end in September 2023.

## Administrative information

**Table:** 

Title {1}	7Adjustment of positive end-expiratory pressure to body mass index during mechanical ventilation in general anesthesia: BodyVent, a randomized controlled trial
Trial registration {2a and 2b}	DRKS00031336, https://drks.de/search/en/trial/DRKS00031336 All items from the WHO Trial Registration Data set can be found within the study protocol or on the DRKS website
Protocol version {3}	28th March 2024. Revised Version
Funding {4}	n/a, external funding not necessary
Author details {5a}	Helene Selpien^1*^ (helene.selpien@uksh.de) Christine Eimer^1*^ (christine.eimer@uksh.de) David Thunecke^1^ (david.thunecke@uksh.de) Jann Penon (jann.penon@uksh.de) Dirk Schädler^1^ (dirk.schaedler@uksh.de) Ingmar Lautenschläger (ingmar.lautenschlaeger@uksh.de) Henning Ohnesorge^1^ (henning.ohnesorge@uksh.de) Tobias Becher^1^ (tobias.becher@uksh.de) Department for Anesthesiology and Intensive Care Medicine, University Hospital Schleswig–Holstein, Campus Kiel Email: helene.selpien@uksh.de
Name and contact information for the trial sponsor {5b}	University Hospital Schleswig–Holstein, Campus Kiel Department for Anesthesiology and Intensive Care Medicine Helene Selpien Arnold-Heller Str. 3, 24105 Kiel, Germany Tel: 0431 500 20801
Role of sponsor {5c}	n/a, no trial sponsor

## Introduction

### Background and rationale {6a}

An increasing number of patients suffer from, partly undiagnosed, pulmonary diseases and other risk factors such as obesity, that pose major challenges to mechanical ventilation. Approximately 5% of patients undergoing surgery under general anesthesia and thus mechanical ventilation develop postoperative pulmonary complications (PPCs). These are associated with higher morbidity and mortality and prolonged hospital stay [1].

Lung-protective ventilation during general anesthesia can help to prevent these PPCs and improve outcomes [2]. Components of lung-protective ventilation include low tidal volumes and adequate positive end-expiratory pressure (PEEP). However, the proper strategy for setting PEEP during general anesthesia is still controversial. Randomized controlled trials comparing higher vs. lower PEEP settings without individualization found no difference in PPCs between higher and lower PEEP [3, 4]. Some evidence suggests that changes in ventilatory settings that result in a reduction in the pressure difference between end-inspiratory plateau pressure and end-expiratory pressure (“driving pressure,” ΔP) may help reduce the risk for PPCs [5]. Previously published approaches for intraoperative individualization of PEEP and reduction of ΔP include adjustment of PEEP according to transpulmonary pressure [6, 7] and performing a decremental PEEP trial and subsequently selecting PEEP according to best respiratory system compliance [7, 8] or with electrical impedance tomography [9, 10]. These methods of individualizing PEEP, however, are not without risk to the patient, are time consuming, and are not easy to implement in routine anesthesiology practice. We therefore sought to develop a method for individual PEEP adjustment that requires neither time-consuming ventilatory maneuvers nor additional equipment.

In a previous study, we found that the PEEP level needed to minimize both end-inspiratory overdistension and alveolar collapse during mechanical ventilation for general anesthesia in flat supine position was positively correlated with the patient’s body mass index and can be estimated using the regression equation PEEP = BMI/3 [11]. In a group of 25 obese patients with an average BMI of 48.3 (BMI/3 = 16.1), Nestler et al. found that the PEEP level required to minimize the regional ventilation delay index (an EIT measure that correlates with tidal recruitment) was 18.5 ± 4.6 mbar and was positively correlated with BMI [9]. In another EIT study investigating the PEEP level required to minimize both end-inspiratory overdistension and alveolar collapse during mechanical ventilation for general anesthesia that included 40 patients (average BMI: 29.5 $\to$ BMI/3 = 9.8), Pereira et al. found an average required PEEP level of 10.3 ml/mbar in the open abdominal surgery group that was also positively correlated with patient’s BMI [10]. In 37 patients scheduled for elective bariatric surgery with an average BMI of 48.3 kg/m^2^ (BMI/3 = 16.1), Eichler et al. found that the target of a positive transpulmonary pressure at end expiration was achieved at PEEP levels of 16.7 cmH_2_O [6].

In summary, these studies suggest that the PEEP needed to prevent both end-inspiratory overdistension of the lung and end-expiratory alveolar collapse during general anesthesia, thereby reducing the ΔP, is positively correlated with the patient’s BMI, with required PEEP levels corresponding on average to approximately one-third of the patient’s respective BMI. We therefore hypothesize that adjusting PEEP to a level corresponding to one-third of the patient’s BMI could lead to a reduction of ΔP and may thus help to prevent PPCs.

### Objectives {7}

The main objective of this study is to evaluate the efficacy of a BMI-adjusted PEEP compared with a standardized PEEP of 5 mbar in reducing the pressure difference between end-inspiratory plateau pressure and end-expiratory pressure. Secondary objectives will examine the difference between intervention and control group concerning respiratory system mechanics, hemodynamics, need for rescue maneuvers, and changes in ultrasound lung aeration score.

### Trial design {8}

The BodyVent trial is a randomized, controlled, patient blinded single-center superiority trial with two parallel groups and a primary endpoint of driving pressure (ΔP) within volume-controlled ventilation in endotracheally intubated patients during general anesthesia. Randomization is performed by random permuted block randomization with a 1:1 allocation ratio.

## Methods: participants, interventions, and outcomes

### Study setting {9}

The study will be performed within the University Medical Center Schleswig–Holstein, Germany’s third-largest university hospital with annually more than 110,000 inpatients. The trial will include patients who will undergo a planned surgery under general anesthesia with endotracheal intubation in the central operating room at Campus Kiel.

### Eligibility criteria {10}

To be eligible, patients must meet the following inclusion criteria: [1] Surgery under general anesthesia with endotracheal intubation is planned, [2] the expected duration of surgery exceeds 2 h, [3] participants are at least 18 years of age, and [4] written informed consent is obtained. Potential patients will be excluded if any of the following exclusion criteria are fulfilled: [1] planned intraoperative ventilation via a face mask or a laryngeal mask airway, [2] laparoscopic surgery, thoracic surgery or surgery with cardiopulmonary bypass is planned, [3] surgery will be performed in > 10° Trendelenburg/Anti-Trendelenburg position, [4] pregnancy, [5] BMI > 60, [6] acute cardiac decompensation, [7] severe pre-existing pulmonary conditions (e.g., acute pulmonary decompensation, acute respiratory distress syndrome, pulmonary fibrosis, present pneumonia, chronic obstructive pulmonary disease severity GOLD IV), [8] inability to give informed consent.

### Who will take informed consent? {26a}

At the pre-medication outpatient clinic patients will be screened daily (Monday–Friday) for possible study inclusion. After detailed information about the BodyVent trial is given, patients will be asked to give written informed consent for the collection of demographic and clinical data and overall study participation by a physician member of the research team. In accordance with the Declaration of Helsinki and national regulations, patients have the right to withdraw their consent at any time and without need to specify reasons without compromising their future medical care.

### Additional consent provisions for collection and use of participant data and biological specimens {26b}

Ancillary studies are currently not planned. The existing declaration of consent also covers possible future projects or the transfer of pseudonymized data to scientific cooperation partners.

## Interventions

### Explanation for the choice of comparators {6b}

In normal weight patients, a PEEP setting of 5 mbar is considered as clinical standard. With an increasing number of overweight patients, a weight-adapted PEEP adjustment may be necessary. To be able to clinically apply optimal and standardized ventilation parameters for individual, weight-adjusted ventilation, an easy-to-apply calculation method is needed. Thus, we consider a comparison between a control group with standard therapy (PEEP 5 mbar) and an intervention group (PEEP BMI/3 mbar) to be useful. The same inclusion and exclusion criteria will be used to recruit the intervention and control group.

### Intervention description{11a}

Only patients who provide consent will be included in the study. After obtaining written informed consent, total height, weight, and waist to hip ratio will be measured. On the day of surgery, a preoperative lung ultrasound examination will be carried out immediately before the patient is admitted to the operating room and the lung aeration score will be calculated as described in [12]. After induction of general anesthesia, patients will be assigned to a group by opening a sealed envelope containing the allocation. All patients will be ventilated in a volume-controlled mode (VCV) with constant inspiratory flow at a tidal volume of 7 ml per kg predicted body weight (PBW), with an I:E ratio of 1:2 and an inspiratory pause of 30%. PBW will be calculated using the NIH-NHLBI ARDS Network formula. ♂: PBW (kg) = 50 + 0.91 (height (cm) − 152.4) and ♀: PBW (kg) = 45.5 + 0.91 (height (cm) − 152.4) [19].

**Fig. 1:**
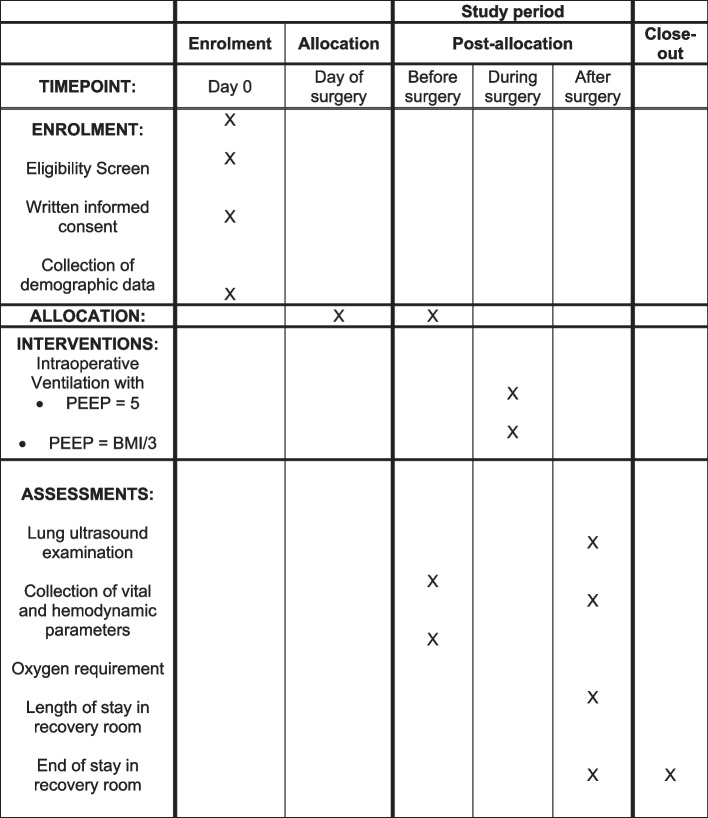
SPIRIT figure: Schedule of enrolment, allocation, interventions, and assessments

In the intervention group, PEEP will be adjusted to a value of BMI/3 mbar for the duration of general anesthesia. In the control group, PEEP will be set at a fixed value of 5 mbar for the duration of general anesthesia. Sustained-inflation recruitment maneuvers will be performed after disconnection of ventilator circuit and in case of a drop in oxygen saturation below 90% despite ventilation with more than 60% of oxygen, with an airway pressure of 20 mbar above PEEP for 10 s in the intervention group and with an airway pressure of 30 mbar for 10 s in the control group. The lung ultrasound data will be stored anonymously and evaluation of the ultrasound images for calculation of lung aeration score will take place without knowledge of the group allocation (Fig. 1).

### Criteria for discontinuing or modifying allocated interventions {11b}

If instances of hypotension or hemodynamic instability necessitating intervention occur, or if the patient experiences acute cardiac or pulmonary decompensation, the intervention will be discontinued. Treating physicians (who are not part of study team) can suspend the study at any time. The reason will be documented in the case report form. Furthermore, participants may withdraw from the study without citing a reason at any time. There is no provision for modifying the assigned intervention.

### Strategies to improve adherence to interventions {11c}

Adherence to the study protocol is ensured by continuous monitoring of the patient throughout the intervention by a member of the research team. This investigator will not only perform the pre- and postoperative sonographic examinations but will also ensure that the intervention is intraoperatively performed correctly by the clinician.

### Relevant concomitant care permitted or prohibited during the trial {11d}

All procedures, interventions, and medications required for optimal patient treatment are allowed during the study. Any medications administered or additional interventions required during the trial will be documented on the case report form. All procedures, interventions, and medications administered in this study adhere to established internal standard procedures and clinical protocols. Potential side effects primarily include hemodynamic or respiratory complications. However, to mitigate these risks, each patient will be under the direct supervision of a board-certified anesthesiologist with extensive experience in managing such complications during surgical procedures. Consequently, we assess the likelihood of experiencing adverse events as low. Detailed information regarding possible adverse events is provided in Item 22. Additionally, as stated in Item 30, patient insurance coverage is available to address any adverse events should they occur.

### Provisions for post-trial care {30}

A patient insurance with an insurance number of maximum 5,000,000 Euro (maximum 500,000 Euro per participant) exists with HDI Global SE (99.9%) and HDI-Haftpflichtverband der Deutschen Industrie V.a.G. (0.1%) if study-associated complications should occur. The monitoring period ends after discharge from the recovery room. Further follow-up is not required.

### Outcomes {12}

The primary endpoint of this trial is the difference in driving pressure (ΔP) between the intervention and control group during general anesthesia in endotracheal intubation and volume-controlled ventilation (VCV). Secondary endpoints include:

- Mechanical Power of ventilation (elastic-dynamic, tidal and total).
- Compliance of the respiratory system.
- Intraoperative fluid requirements.
- Average intraoperative vasopressor requirements (µg/kg/min).
- Number of intraoperative hypotension events (MAD < 65 mmHg for > 1 min).
- Time-weighted average of hypotension.
- Number of alveolar recruitment maneuvers (to be performed if SpO_2_ < 90% with FiO_2_ > 60%).
- SpO_2_ after arrival in recovery room (without oxygen insufflation).
- Number of patients with indication for oxygen insufflation (to maintain SpO_2_ ≥ 90%).
- Change in lung aeration score between before surgery and after arrival in recovery room(determined by lung ultrasound).
- Overall postoperative pulmonary complications.

For further clarification see, Table 1 (“Outcome Definition”).

**Table 1:** Outcome definition

**Domain**	Respiratory mechanics	Rescue therapy	Hemodynamics	Postoperative status
Specific measures	• Driving pressure • Mechanical power of ventilation (elastic-dynamic, tidal, and total) • Compliance of respiratory system	• Number of alveolar recruitment maneuvers	• Intraoperative fluid requirements • Average intraoperative vasopressor requirements • Number of intraoperative hypotension events • time-weighted average of hypotension	• SpO_2_ after arrival in recovery room • Number of patients with additional oxygen insufflation^a^ • Change in lung aeration score
Specific metric	Difference between control and intervention group	Difference between control and intervention group	Difference between control and intervention group	Difference between control and intervention group
Method of aggregation	Mean + SD (parametric data) or Median/IQR (non-parametric data)	Mean + SD (parametric data) or Median/IQR (non-parametric data)	Mean + SD (parametric data) or Median/IQR (non-parametric data)	Mean + SD (parametric data) or Median/IQR (non-parametric data) and four-square table^a^
Time point	Intraoperative	Intraoperative	Intraoperative	Postoperative

Outcomes are defined following the framework of Saldanha et al. [20]

^a^Four-square table is only used as method of aggregation for “number of patients with additional oxygen insufflation”

### Participant timeline {13}

**Fig. 2:**
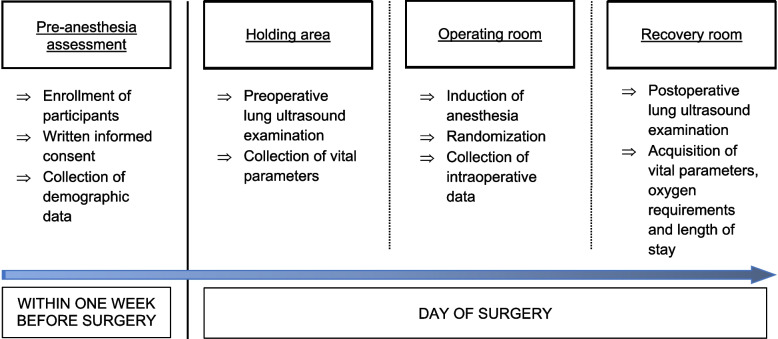
Patient timeline showing the different steps of the study process

Within the week before surgery, patients will visit the pre-medication outpatient clinic. Participants will be screened and, if eligible, will be asked to provide written informed consent to participate in the study. Only patients who provide consent will be included in the study. After obtaining consent, total height, weight, and waist to hip ratio will be measured. On the day of surgery, a preoperative lung ultrasound examination will be carried out immediately before the patient is admitted to the operating room. After induction of general anesthesia, patients will be randomized by opening a sealed envelope. The protocol is then carried out accordingly for the control group or for the intervention group. After extubation, patients will be transferred to the recovery room. A postoperative lung ultrasound examination will be carried out. Additional oxygen will be applied if oxygen saturation drops below 90% breathing room air. Patients will be discharged from the recovery room according to routine clinical criteria (oxygen saturation > 90% or similar to preoperative value breathing room air, hemodynamic stability, patient awake and oriented, sufficient pain control). Data collection ends after patient is discharged; no further follow-up is needed (Fig. 2).

### Sample size {14}

Sample size calculation was performed with G*power 3.1 Software [13]. Assuming a mean (± SD) ΔP of 10 (± 3) mbar at PEEP = 5 mbar and a mean ΔP of 8 (± 2) mbar at PEEP = BMI/3 mbar, *α* = 0.05, 1 − *β* = 0.8, G*Power yielded a required sample size of *n* = 54 (27 per group) patients. The anticipated effect size is based on the results previously published by Pereira et al. [10]. To compensate for refusal, technical difficulties, or possible dropouts, we will increase the sample size by 10%, resulting in a total sample size of 60 patients.

### Recruitment {15}

Recruitment in the pre-medication outpatient clinic will be continuously conducted by a member of the research team until the target randomized sample size of 60 participants is achieved. Based on current clinical case numbers, this will take approximately 6 months.

## Assignment of interventions: allocation

### Sequence generation {16a}

Randomization is performed by permuted block randomization with a 1:1 allocation ratio. Randomly permuted blocks of varying sizes will be used. The order within each block and the block order itself are randomly determined by one member of the research team with the help of www.randomizer.org. Said researcher will not participate in patient recruitment or data collection. All other research team members and participants are blinded to the randomization sequence.

### Concealment mechanism {16b}

To ensure that allocation is unbiased and concealed from patients and investigators, a numbered opaque envelope containing the group assignment is opened after induction of anesthesia by a data collecting member of the research team.

### Implementation {16c}

The allocation sequence is generated by an independent member of the research team who is not involved in screening or analysis. In envelopes prepared by the independent investigator, randomization is already established as described above and will be implemented after induction of anesthesia. Enrollment, clinical data collection, implementation to assigned allocation group, and monitoring of the procedure is performed by a data collection team.

## Assignment of interventions: blinding

### Who will be blinded {17a}

Trial participants will remain blinded to their randomized group assignment. However, due to the nature of the intervention, blinding is not feasible for clinicians performing the intervention, data collectors overseeing its implementation, and data analysts analyzing ventilator data, as readouts of respiratory and hemodynamic parameters inherently contain the implemented PEEP. Consequently, hemodynamic and respiratory data will be analyzed by non-blinded members of the research team. Given that this data is recorded on an ordinal scale, it is neither subject to interpretation nor distortion. The investigators evaluating the lung ultrasound data, on the other hand, will be blinded to the patient’s group allocation.

### Procedure for unblinding if needed {17b}

The data collection team is encouraged to maintain blinding as far as possible. The allocation will not be disclosed to the patient and to the lung ultrasound data analyzing team; nor will there be any written or verbal disclosure of the code in any of the corresponding non-pseudonymized patient documents. As the treating anesthesiologists are not blinded to group allocation, there is no need to establish an unblinding procedure.

## Data collection and management

### Plans for assessment and collection of outcomes {18a}

Demographic data such as height, weight, and waist to hip ratio, as well as clinical data, which includes medication given, ventilation parameters, and vital data, are recorded on paper-based case report forms (CRFs, see appendix) by data collectors. Additionally, ventilator measurement data are exported electronically from the ventilator and hemodynamic data are exported electronically from the Philips monitoring. By duplicating the data collection (paper based + electronic), participation can be ensured even in the event of technical transmission or recording problems. Data collected on paper forms will be entered and then double checked by an independent researcher to ensure reliability and accuracy of the data.

To increase reliability and validity of the ultrasound examination, members of the research team will be trained in lung ultrasonography and solely these investigators will perform the examinations using a 1.5–6 MHz curved array probe (Vivid S70 N, GE Healthcare). To determine the secondary outcome “change in lung aeration score between pre- and postoperative lung ultrasound examination,” ultrasound data will be assessed by two trained investigators without knowledge of group allocation using the lung aeration score modified for perioperative setting [12]. The investigators are also trained prior to assessment to achieve optimal reliability and validity. To determine the primary and further secondary outcome, ventilator data and hemodynamic data will be analyzed by the data analyzing team as described below (20a).

### Plans to promote participant retention and complete follow-up {18b}

N/a. No follow-up for clinically relevant outcome measurement needed.

### Data management {19}

All participant information is stored in locked file cabinets and password-protected databases to which only research team members have access. Collected data are pseudonymized by a coded ID [identification] number. All records that contain names or other personal identifiers, such as informed consent forms, will be stored separately from study records identified by code number. Demographic and clinical data, as well as information on hypotension events and postoperative oxygen requirements, are recorded on paper-based case report forms (CRFs) and stored in locked file cabinets. Log files containing ventilator data are exported from ventilators and saved on a password-secured network drive. Hemodynamic data are exported from the Philips monitoring and secured on the same network drive. Ultrasound data are pseudonymized and stored on the ultrasound equipment and subsequently transferred to a password-protected network drive for further analysis. The clinical data will not only be recorded digitally, but also handwritten in case of possible technical problems or study protocol breaches.

### Confidentiality {27}

Data are handled confidentially, and transfer of patient-related medical data is pseudonymized. No features are transferred that allow direct identification of specific participants. The subject identification code list to personal data is accessible only to the principal investigator. All further records containing names or other personal identifiers, such as informed consent forms, are kept separate from the study data identified by code number. Data collection, coding, security, and storage will comply with the provisions of the German Federal Data Protection Act (BDSG) and the EU General Data Protection Regulation (EU-GPDR). Accordingly records and documents related to the clinical trial will be kept for at least 15 years.

### Plans for collection, laboratory evaluation, and storage of biological specimens for genetic or molecular analysis in this trial/future use {33}

N/a. No biological specimens will be collected.

## Statistical methods

### Statistical methods for primary and secondary outcomes {20a}

The primary objective of the study is to determine whether BMI-adjusted ventilation strategies result in a reduction of driving pressure. Data are tested for normal distribution using the Shapiro–Wilk test. Differences between the intervention group and the control group are assessed with a two-tailed unpaired *t*-test for normally distributed continuous data and with the Mann–Whitney test for non-normally distributed continuous data. In addition, descriptive statistical analyses (mean ± standard deviation, median, interquartile range, and 95% confidence interval, if applicable) will be performed.

For secondary endpoints, to evaluate the changes in lung ultrasound score between the pre- and postoperative groups and between the intervention groups, a two-way ANOVA will be performed with the time point (preoperative/postoperative) as one independent variable and the study group (intervention/control) as the other independent variable. For categorical variables (hypotension events, number of patients requiring supplemental oxygen), Fisher’s exact test will be used. For the secondary endpoint “time-weighted average of hypotension events,” the area under the threshold of 65 mmHg (AUC) for each event will be calculated by summing the difference between MAP and the threshold, multiplied by the duration of the event. Total AUC will be divided by the observed time, which varies by patient. This results in a time-weighted average of hypotension per patient.

### Interim analyses {21b}

Not applicable, there will be no interim analyses.

### Methods for additional analyses (e.g., subgroup analyses) {20b}

Not applicable, further analyses are not planned.

### Methods in analysis to handle protocol non-adherence and any statistical methods to handle missing data {20c}

If the protocol could not be completely adhered to, or individual data is missing, a drop-out rate of 10% was considered in the sample size calculation. A per-protocol analysis will be performed with respect to the study intervention (PEEP setting). Data sets will only be excluded from the per-protocol analysis if the study intervention (PEEP setting) was not performed, or if the primary endpoint (ΔP) cannot be evaluated. For all other endpoints, if there are missing values, the number of missing values will be reported in the manuscript and analyses will be performed as planned.

### Plans to give access to the full protocol, participant-level data, and statistical code {31c}

Datasets containing anonymized patient data will be made accessible upon reasonable request to the corresponding author, in compliance with European data protection regulations (GDPR).

## Oversight and monitoring

### Composition of the coordinating centre and trial steering committee {5d}

The principal investigator is responsible for the monitoring of the study process. The study team consists of the principal investigator, a deputy investigator, and several sub-investigators and two study nurses. The group meets once every 2 weeks to discuss different issues and monitor the progress of the trial. The progression of the study, the presence of informed consent, review of the adherence to the study protocol, and verification of accuracy and completeness of the data will be checked by the principal investigator, the deputy investigator, and the two study nurses.

### Composition of the data monitoring committee, its role and reporting structure {21a}

This single-center pilot study is not powered to investigate patient-centered outcome parameters. We expect minimal risk from the study intervention. Therefore, no interim analyses are planned or will be performed, and a data monitoring committee has not been organized for this study.

### Adverse event reporting and harms {22}

Adverse events and other unintended effects of the trial will be collected, assessed, and immediately reported to the principal investigator. Potential harms and complications encompass instances of hypotension or hemodynamic instability necessitating intervention, as well as occurrences such as barotrauma, dislocation of the endotracheal tube, or peripheral oxygen saturation dropping below 92%. However, monitoring of patients will be conducted throughout their participation in the study, with treatment provided for any complications that arise. Incidences will be electronically recorded according to the study protocol and subsequently reported in the “Results” section.

### Frequency and plans for auditing trial conduct {23}

Not applicable, an auditing trail conduct is not required.

### Plans for communicating important protocol amendments to relevant parties (e.g., trial participants, ethical committees) {25}

Any changes in protocol will be submitted to the local ethics committee for approval, all changes will be communicated to the study team via email and during the regular study group meetings. The trial record on DRKS will be updated accordingly.

### Dissemination plans {31a}

Once the study has been completed, the results will be published in a peer-reviewed scientific journal and presented on national and international conferences for anaesthesiology and intensive care medicine.

## Discussion

This study aims to evaluate whether intraoperative PEEP adjustment according to BMI is safe, reduces driving pressure by increasing respiratory system compliance, and prevents loss of lung aeration as assessed by lung ultrasound in comparison to a standardized PEEP setting of 5 mbar.

Induction of general anesthesia and the subsequent loss of spontaneous respiration result in decisive changes in lung physiology, leading to atelectasis, reduced gas exchange, and reduced end-expiratory lung volume. These effects are partly dependent on body weight and might be more pronounced in obese patients, indicating the need for individualized ventilation strategies [9]. Nevertheless, the individualized approach must still be clinically feasible. Although the results may not be as ideal as with more elaborate methods of PEEP adjustment such as electrical impedance tomography or transpulmonary pressure measurement, a simple tool such as the rule of thumb PEEP = BMI/3 could represent a clinically feasible compromise between individualization and applicability.

Lung ultrasound has found widespread integration in clinical routine regarding the assessment of lung aeration in patients with dyspnea and acute respiratory failure [14] and is an established and readily available noninvasive method tool for the detection of postoperative pulmonary complications. Lung ultrasound allows early detection of changes in lung structure that are typical of PPCs, such as an increase in lung density due to atelectasis or effusions [15]. However, visualization of a single examination cannot be considered specific enough for diagnosis, thus it is deemed useful to perform an initial examination preoperatively before intubation and a follow-up examination postoperatively after termination of mechanical ventilation [12]. To establish longitudinal comparability and classify the severity of lung structural change, a standardized lung ultrasound scoring system should be applied [16]. The lung aeration score, based on the BLUE protocol [14], represents a validated score that has been successfully applied several times. Sonography is performed on spontaneously breathing patients in six regions per hemithorax, 0 to 3 points are assigned to each region according to the observed lung ultrasound pattern, and a sum is calculated. To evaluate the immediate postoperative outcome, oxygen saturation, required oxygen insufflation, and duration of stay in the recovery room will be analyzed as secondary outcome parameters. The length of stay in the recovery room will usually be half of the operating time and can be terminated as soon as the patient is hemodynamically and respiratory stable without further interventions.

To ensure reliable data collection and analysis, the team collecting the data was trained in the performance of lung ultrasound and the analysis team was trained in the use of the lung ultrasound score before the start of the study, using 20 sample patients. Therefore, to reduce examiner bias and prevent potential sources of error, acquisition and assessment of ultrasound images will always be performed by at least two different researchers. Lung ultrasound analyzers are blinded to group assignment and other clinical data.

Several previously conducted studies propose an association between high ΔP, high elastic-dynamic mechanical power (the product of ΔP, tidal volume, and respiratory rate) and unfavorable outcomes for patients with acute respiratory distress syndrome [17, 18]. For patients undergoing mechanical ventilation during general anesthesia, the available evidence suggests that a ventilation strategy that leads to a reduction in ΔP also reduces the risk of postoperative pulmonary complications [5]. For this reason, ΔP was chosen as primary endpoint in this trial. To further analyze the effect of PEEP adjustment according to BMI, respiratory system compliance, the different components of mechanical power (elastic-static, elastic-dynamic, resistive, total, tidal), intraoperative hemodynamics, postoperative oxygen requirements, and the number of intraoperative recruitment maneuvers required to maintain SpO_2_ > 90% with FiO_2_ < 60% will be recorded.

Available data suggest that 1 mbar increase in ΔP leads to a 16% relative risk increase for postoperative pulmonary complications (odds ratio for one unit increase of driving pressure 1.16) [5]. With an incidence of postoperative pulmonary complications around 5% in a mixed surgical population [1] and an assumed reduction of driving pressure by 1–2 mbar with PEEP adjusted according to BMI, a sample size of several thousand patients would be required to show a statistically significant reduction in postoperative pulmonary complications with our strategy. We therefore chose driving pressure as easy to measure and clinically relevant surrogate outcome parameter for our single-center trial assuming that a ventilation strategy that leads to a reduction in ΔP will ultimately reduce the risk of postoperative pulmonary complications in larger populations.

However, as this is the subject of the study, we cannot exclude with certainty that ΔP and the associated risk of postoperative pulmonary complications could also be increased with the ventilation strategy studied. Further limitations include that results may not be applicable to procedures with altered intrathoracic or intraabdominal pressures, such as laparoscopic or thoracic procedures or patient’s positions other than flat supine. Due to the study design the occurrence of PPCs could not be evaluated, this would have required a significantly higher number of patients. Thus, this aspect should be considered in further studies.

In conclusion, the study results will show whether an intraoperative ventilation strategy with PEEP adjustment based on BMI has the potential of reducing the risk for postoperative pulmonary complications as an easy-to-implement intervention that does not require lengthy ventilator maneuvers nor additional equipment.

## Trial status

The study protocol (version 1.1, date 31st January 2023) was approved by the ethics committee of the Christian-Albrechts-Universität Kiel, Germany, on 1st February 2023. Recruitment began on 3rd March 2023. Planned study completion date 30th September 2023.

## Data Availability

Following publication of the trial, datasets containing pseudonymized patient data will be made accessible upon reasonable request to the corresponding author.

## Abbreviations

ANOVA: Analysis of variance
AUC: Area under curve
BDSG: German Federal Data Protection Act
BMI: Body mass index
CRF: Case report form
EU-GDPR: European Union General Data Protection Regulation
PEEP: Positive end-expiratory pressure
*P*_insp_: Inspiratory pressure
PPC: Postoperative pulmonary complication
SpO_2_: Oxygen saturation
ΔP: Driving pressure
